# Induction of Mast Cell Accumulation by Tryptase via a Protease Activated Receptor-2 and ICAM-1 Dependent Mechanism

**DOI:** 10.1155/2016/6431574

**Published:** 2016-06-09

**Authors:** Xin Liu, Junling Wang, Huiyun Zhang, Mengmeng Zhan, Hanqiu Chen, Zeman Fang, Chiyan Xu, Huifang Chen, Shaoheng He

**Affiliations:** ^1^Allergy and Clinical Immunology Research Centre, The First Affiliated Hospital of Jinzhou Medical University, Jinzhou, Liaoning 121001, China; ^2^Allergy and Inflammation Research Institute, The Key Immunopathology Laboratory of Guangdong Province, Shantou University Medical College, Shantou 515031, China

## Abstract

Mast cells are primary effector cells of allergy, and recruitment of mast cells in involved tissue is one of the key events in allergic inflammation. Tryptase is the most abundant secretory product of mast cells, but little is known of its influence on mast cell accumulation. Using mouse peritoneal model, cell migration assay, and flow cytometry analysis, we investigated role of tryptase in recruiting mast cells. The results showed that tryptase induced up to 6.7-fold increase in mast cell numbers in mouse peritoneum following injection. Inhibitors of tryptase, an antagonist of PAR-2 FSLLRY-NH_2_, and pretreatment of mice with anti-ICAM-1, anti-CD11a, and anti-CD18 antibodies dramatically diminished tryptase induced mast cell accumulation. On the other hand, PAR-2 agonist peptides SLIGRL-NH_2_ and tc-LIGRLO-NH_2_ provoked mast cell accumulation following injection. These implicate that tryptase induced mast cell accumulation is dependent on its enzymatic activity, activation of PAR-2, and interaction between ICAM-1 and LFA-1. Moreover, induction of trans-endothelium migration of mast cells* in vitro* indicates that tryptase acts as a chemoattractant. In conclusion, provocation of mast cell accumulation by mast cell tryptase suggests a novel self-amplification mechanism of mast cell accumulation. Mast cell stabilizers as well as PAR-2 antagonist agents may be useful for treatment of allergic reactions.

## 1. Introduction

Mast cell tryptase belongs to serine proteases and is almost exclusively located to the secretory granules of mast cells. They are the most abundant protein products in mast cell granules, which consist of approximately 50% total protein in the granules [1]. Upon degranulation, tryptase is released from mast cells along with histamine, heparin, chymase, and other mast cell granule products [2]. Large quantities of active form tryptase in mast cells [3] and increased expression of tryptase in the airway of asthma [4] imply that this mast cell unique mediator may contribute to mast cell related airway diseases.

It has been found that tryptase is capable of provoking microvascular leakage in the skin of guinea pigs [5], stimulating the release of histamine from dispersed human tonsil mast cells [6], and inducing recruitment of inflammatory cells to endothelium [7] and eosinophils and neutrophil in peritoneum of mice [8]. These observations implicate that this mast cell protease is likely to play a role in the pathogenesis of mast cell associated inflammation.

Protease activated receptor (PAR) have been identified as receptors for serine proteases. Among them, PAR-1 is a receptor of thrombin and trypsin [9], and PAR-2 is a receptor of trypsin and tryptase [10]. Upregulation of PAR-2 expression in the airways of asthma [11] suggests involvement of PAR-2 in the disease, whereas activation of PAR-2 on mast cells by tryptase [12] implicates a novel self-amplification mechanism of mast cell activation [13]. However, little is known of contribution of tryptase to recruitment of mast cells.

Since recruiting mast cells in involved tissue is one of the key events in the pathogenesis of allergy, mast cell granule product histamine can provoke chemotaxis of mouse mast cells through histamine H_4_ receptor [14], and mast cell product platelet-activating factor (PAF) is capable of inducing a chemotactic response of mast cells [15], we anticipated that tryptase may also have ability to recruit mast cells. Therefore, the aim of the present study is to investigate effects of tryptase on mast cell accumulation and its potential mechanisms.

## 2. Materials and Methods

### 2.1. Reagents

The following compounds were purchased from Sigma-Aldrich (St. Louis, MO, USA): leupeptin, aprotinin, benzamidine, protamine, trypsin, compound 48/80, terfenadine, sodium cromoglycate and human serum albumin (HSA), L-glutamine, hydrocortisone, epidermal growth factor (EGF), penicillin/streptomycin, and N-formyl-methionyl-leucyl-phenylalanine (fMLP). Recombinant human *β* tryptase (rTryptase) was purchased from Promega (Wisconsin, USA). Agonist peptides of protease activated receptor-2 (PAR-2), SLIGRL-NH_2_, and trans-cinnamoyl (tc-) LIGRLO-NH_2_ as well as their reverse forms LRGILS-NH_2_ and tc-OLRGIL-NH_2_ and PAR-2 antagonist peptide FSLLRY-NH_2_ were synthesized by CL Bio-Scientific Inc. (Xi An, China) with a purity >98% assessed by HPLC analysis. MCDB 131 medium, RPMI 1640 medium, fetal bovine serum (FBS), MEM containing 25 mM HEPES, and Dulbecco's Phosphate-Buffered Saline (DPBS) were obtained from Invitrogen-Gibco®/Life Technologies (Grand Island, NY, USA). Rat monoclonal antibodies including anti-mouse CD11a [lymphocyte function-associated antigen 1 (LFA-1) *α* chain], anti-mouse CD18 (integrin *β*2 chain), anti-mouse CD 62L (L-selectin), rat IgG2a isotype standard, hamster anti-mouse CD54 [intercellular adhesion molecule 1 (ICAM-1)] antibody and hamster IgG1 isotype standard, clone A19-3, and mouse anti-human CD54 monoclonal antibody, clone HA58, were purchased from BD Biosciences Pharmingen (Bedford, MA, USA). PE conjugated anti-human CD117 antibody was obtained from Biolegend (San Diego, USA). Serotonin ELISA kit was purchased from Abnova (Taiwan, China). Modified Wright's stain was from BaSo (Zhuhai, China).

### 2.2. Animals, Cell Line, and Culture

BALB/c mice (18–22 g) were obtained from Guangdong Experimental Animal Centre, China, Grade II, Certificate number 2001A049, and from Vital River Laboratory Animal Technology Co. Ltd. (Beijing, China), Certificate number 11400700056942. The animal experiment procedures were approved by the Animal Care Committee at Shantou University and Jinzhou Medical University. Human mast cell line, HMC-1 cells, was a present from Dr. Joseph H. Butterfield (Mayo Clinic, MN, USA). HMC-1 cells were maintained in RPMI 1640 medium supplemented with 10% (v/v) heat-inactivated FBS, 100 units/mL penicillin/streptomycin in 75 cm^2^ tissue culture flasks (Falcon) at 37°C in a humidified atmosphere of 5% (v/v) CO_2_. HMEC-1 (ATCC® CRL-3243*™*, human dermal microvascular endothelium) cells were cultured in MCDB 131 medium containing 2.0 mM glutamine, 1.0 *μ*g/mL hydrocortisone, and 10 ng/mL epidermal growth factor supplemented with 10% (v/v) heat-inactivated FBS, 100 units/mL penicillin/streptomycin. When HMEC-1 reached 80% confluence in culture flasks, the recommended trypsin-EDTA solution was used to disperse the cells and the cells were used in experiments or reseeded in flask.

### 2.3. Preparation of Compounds

Tryptase was purified from human lung and skin tissues by high salt extraction, heparin agarose, and immunoaffinity chromatography procedures with monoclonal antibody AA5 against tryptase as described previously [8]. The specific activity of the tryptase used in these studies was 1.96 and 1.84 U/mg for lung tryptase (lTryptase) and skin tryptase (sTryptase), respectively. The preparation had no detectable chymotryptic or elastolytic activity, and endotoxin levels were very low, being less than 49 pg/mg tryptase.

As tryptase is enzymatically unstable in physiological solutions, considerable care was taken in its preparation. Purified tryptase stored in high salt buffer was diluted immediately prior to its injection, first with sterile distilled water, adjusting the NaCl concentration to 0.15 mol·L^−1^, and then with normal saline to obtain the required tryptase concentration. HSA in saline was used as a foreign protein control. Where added, proteinase inhibitors were incubated with lTryptase for 30 min on ice before injection. For the heat inactivation, lTryptase was heated at 56°C for 120 min.

### 2.4. Mouse Peritoneal Injection and Cell Count

The procedure was adapted from that described previously [8]. Briefly, various concentrations of lTryptase and sTryptase, rTryptase, compound 48/80, SLIGRL-NH_2_, LRGILS-NH_2_, tc-LIGRLO-NH_2_, tc-OLRGIL-NH_2_, and HSA, leupeptin at 10 *μ*g/mL, aprotinin at 10 *μ*g/mL, benzamidine at 10 *μ*g/mL, antipain at 10 *μ*g/mL, protamine at 10 *μ*g/mL, *α*1-antitrypsin at 10 *μ*g/mL, PAR-2 antagonist peptide FSLLRY-NH_2_ at 400 *μ*M, HSA (as a foreign protein control), or normal saline were injected in 0.5 mL volumes into the peritoneum of mice, whose abdominal skin was swabbed with 70% ethanol. At 10 min, 3 h, 6 h, or 16 h following injection, animals were killed, and their peritoneal lavage fluids were collected into heparinised tubes and centrifuged at 1200 rpm for 10 min at 4°C. Cells were resuspended in 2.0 mL MEM, stained with 0.1% trypan blue, and enumerated using an Improved Neubauer Haemocytometer (for total cell numbers). Cytocentrifuge preparations were made, air dried, and stained with modified Wright's stain. Differential cell counts were performed for a minimum of 500 cells. The results were expressed as absolute numbers of mast cells per mouse peritoneum.

### 2.5. Pretreatment of Mice with Antibodies and Drugs

For the experiments investigating mast cell migration mechanism, groups of mice were pretreated intravenously (tail vein injection) with monoclonal antibodies against the adhesion molecules L-selectin (CD62L), CD11a/CD18, and ICAM-1 (all at a dose of 1 mg·kg^−1^) [16], respectively, for 30 min before intraperitoneal injection of 1.0 *μ*g/mL of lTryptase. Control animals received an equivalent dose of the corresponding normal rat or hamster IgG isotype control alone. At 6 h following injection, the mice were sacrificed and their peritoneal lavages were processed as described above.

### 2.6. Trans-Endothelial Migration of Mast Cells* In Vitro*

To further investigate effect of tryptase on mast cell migration, a coculture system with HMC-1 and HMEC-1 cells was established. Migration of HMC-1 cells was assessed by using E-16-well plates and the xCELLigence technology (Acea Bioscience, San Diego, CA, USA) [17]. Briefly, 165 *μ*L medium solutions containing tryptase at 0, 0.3, and 1.0 *μ*g/mL with or without leupeptin (1.0 *μ*g/mL) were added in lower chamber of E-16-well plates, respectively, and incubated for 1 h. For the upper chamber, 30 *μ*L HMEC-1 cells (6 × 10^4^ cells/well) were seeded in, and cells grew for 1 h. Anti-human ICAM-1 antibody (50 *μ*L) was then added in specified wells and cultured for 15 min. This was followed by seeding 50 *μ*L HMC-1 cell suspension (6 × 10^4^ cells/well). All control wells received only the equal volume of medium. The HMC-1 migration number, expressed as a cell index value, was monitored for 48 h. Cells in the lower chamber were collected for flow cytometric analysis of HMC-1. The experiments were conducted in duplicate and repeated 4 times.

### 2.7. Flow Cytometry Analysis of Mast Cells and Endothelial Cells

Cells in lower chambers were collected in 1 mL of 1% BSA/PBS and pelleted by centrifugation. This was followed by incubation of cells with PE conjugated anti-human CD117 antibody at room temperature for 15 min in the dark. PE conjugated mouse IgG1 was used as isotype control. After washing, cells were resuspended in fluorescence-activated cell sorting (FACS-) flow solution and analyzed with FACS Verse flow cytometer with CellDevia software (BD Biosciences, San Jose, CA).

### 2.8. ELISA

Levels of serotonin were measured by using ELISA kits according to the manufacturer's instruction.

### 2.9. Statistics

Statistical analyses were performed by using SPSS software (version 17.0, IBM Corporation). Data are displayed as a boxplot, which indicates the median, interquartile range and the largest and smallest values for the number of experiments indicated. Where Kruskal-Wallis analysis indicated significant differences between groups, for the preplanned comparisons of interest, the paired Mann-Whitney *U* test was employed. Data for trans-endothelial migration of HMC-1 cells were expressed as mean ± SEM. Where analysis of variance indicated significant differences between groups with ANOVA, Student's *t*-test was applied. For all analyses, *P* < 0.05 was considered statistically significant.

## 3. Results

### 3.1. Mast Cell Accumulation Induced by Compound 48/80

**Figure 1:**
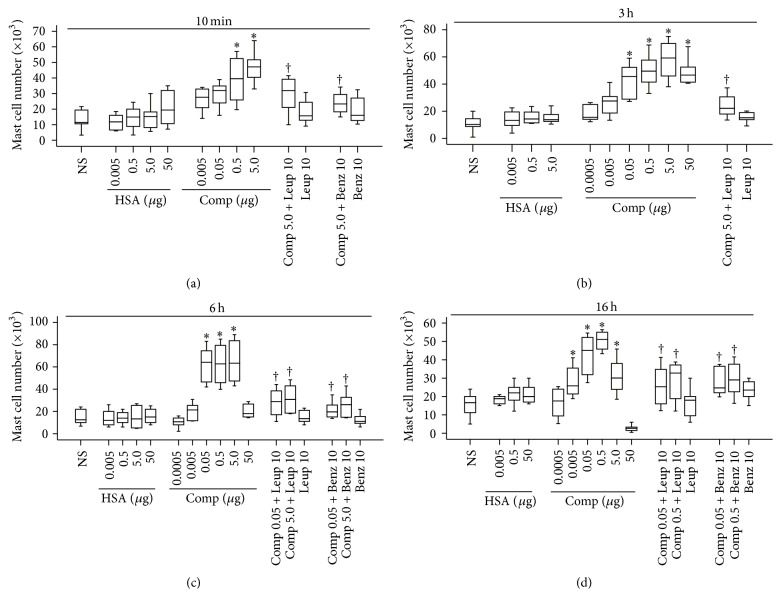
Induction of mast cell accumulation in peritoneum of mice by compound 48/80 (Comp, *μ*g). Various concentrations of Comp in the presence or absence of leupeptin (Leup, *μ*g) and benzamidine (Benz, *μ*g) were injected in peritoneum of mice for 10 min (a), 3 h (b), 6 h (c), and 16 h (d). Human serum albumin (HSA) was used for foreign protein control, and normal saline (NS) was employed as carrier. Data were displayed as a boxplot, which indicates the median, interquartile range and the largest and smallest values. Each piece of data represented a group of 6-7 animals. ^*∗*^ *P* < 0.05 compared with the corresponding NS group. ^†^ *P* < 0.05 compared with the corresponding stimulus alone group.

It was reported that histamine was able to induce chemotaxis of mast cells through histamine H_4_ receptor [14], suggesting a self-amplification mechanism of mast cell accumulation. To further confirm the concept that mast cell degranulation may provoke mast cell accumulation* in vivo*, we investigate the ability of a potent mast cell secretagogue compound 48/80 in induction of mast cell accumulation in the peritoneum of mice. The result showed that compound 48/80 was able to markedly enhance mast cell numbers in mouse peritoneum at 10 min (Figure 1(a)), 3 h (Figure 1(b)), 6 h (Figure 1(c)), and 16 h (Figure 1(d)) following injection. As little as 0.05 *μ*g of compound 48/80 was capable of inducing up to approximately 4.1-fold increase in mast cell accumulation in mouse peritoneum at 6 h following injection. It was observed that tryptase inhibitors leupeptin and benzamidine inhibited compound 48/80 induced mast cell accumulation by up to 69.8% and 82.6% at 6 h following injection, respectively, implicating that the major mast cell granule product tryptase may contribute to compound 48/80 induced mast cell accumulation.

### 3.2. Induction of Mast Cell Accumulation by Tryptase

**Figure 2:**
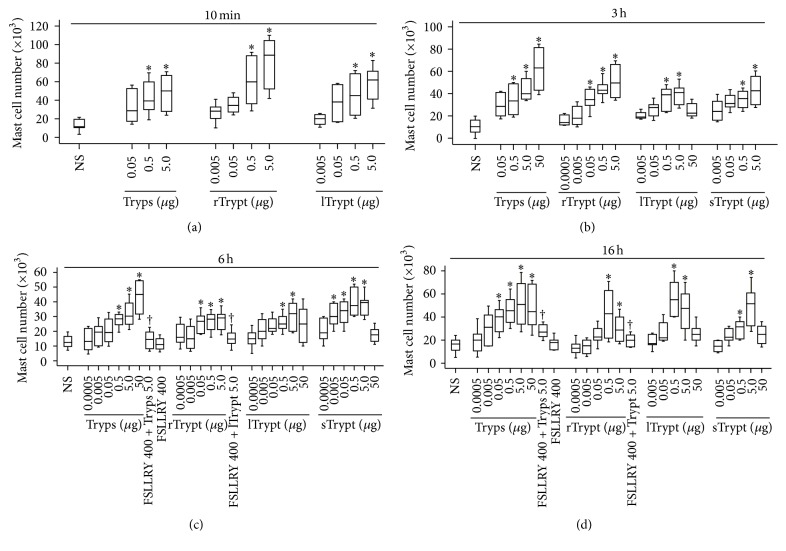
Induction of mast cell accumulation in peritoneum of mice by tryptase. Various concentrations of recombinant tryptase (rTrypt, *μ*g), lung tryptase (lTrypt, *μ*g), skin tryptase (sTrypt, *μ*g), and trypsin (Tryps, *μ*g) in the presence or absence of FSLLRY-NH_2_ (FSLLRY, *μ*M) were injected in peritoneum of mice for 10 min (a), 3 h (b), 6 h (c), and 16 h (d). Normal saline (NS) was employed as carrier. Data were displayed as a boxplot, which indicates the median, interquartile range and the largest and smallest values. Each piece of data represented a group of 6-7 animals. ^*∗*^ *P* < 0.05 compared with the corresponding NS group. ^†^ *P* < 0.05 compared with the corresponding stimulus alone group.

To confirm whether tryptase is capable of eliciting mast cell accumulation, three different sources of tryptase were employed to examine their actions in mast cell accumulation. The results showed that tryptase and trypsin were able to markedly enhance mast cell numbers in mouse peritoneum at 10 min (Figure 2(a)), 3 h (Figure 2(b)), 6 h (Figure 2(c)), and 16 h (Figure 2(d)) following injection. Noticeably, lTryptase, sTryptase, and rTryptase induced dose dependent mast cell accumulation at 6 h following injection. Up to 6.7-fold increases in mast cell numbers were observed when 5.0 *μ*g rTryptase was injected into mouse peritoneum for 10 min. As little as 0.005 *μ*g of tryptase was capable of inducing significant mast cell accumulation in mouse peritoneum at 6 h following injection. Similarly, trypsin induced up to 5.1-fold mast cell accumulation in mouse peritoneum at 3 h.

### 3.3. The Effect of Inhibitors of Tryptase and PAR-2 Antagonists on Tryptase Induced Mast Cell Accumulation

Inhibitors of tryptase including leupeptin, benzamidine, aprotinin, antipain, and protamine suppressed tryptase induced mast cell accumulation by up to 91.2, 84.6, 87.4, 90.1, and 81.2%, respectively, whereas heat-inactivating tryptase (56°C for 120 min) abolished up to 91.1% its ability to accumulate mast cells (Table 1). An antagonist of PAR-2 FSLLRY-NH_2_ inhibited tryptase induced mast cell accumulation by 91.4% at 16 h following injection (Figure 2(d)). Similarly, FSLLRY-NH_2_ at 400 *μ*M diminished approximately 70% trypsin-induced mast cell accumulation at 16 h following injection (Figure 2(d)). These inhibitors by themselves did not alter number of mast cells in the peritoneum of mice when being injected alone (data not shown).

### 3.4. Effect of Agonists of PAR-2 on Mast Cell Accumulation

**Figure 3:**
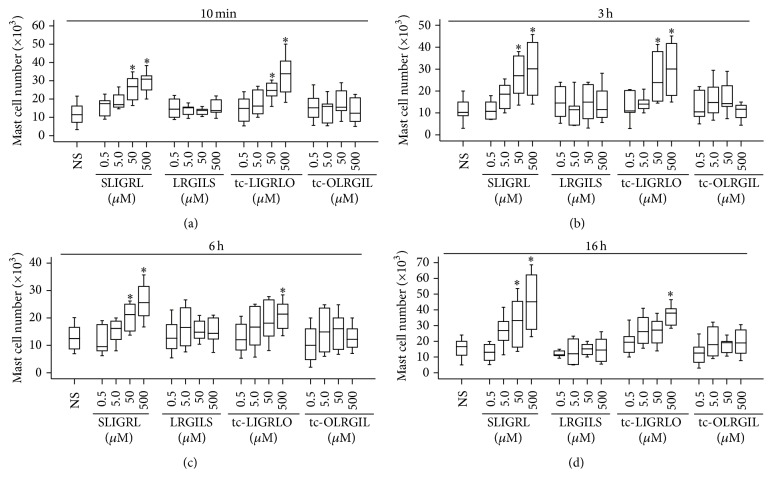
Induction of mast cell accumulation in peritoneum of mice by agonist peptides of protease activated receptor-2 (PAR-2). Various concentrations of SLIGRL-NH_2_ (SLIGRL) and tc-LIGRLO-NH_2_ (tc-LIGRLO) and their reverse peptides LRGILS-NH_2_ (LRGILS) and tc-OLRGIL-NH_2_ (tc-OLRGIL) were injected in peritoneum of mice for 10 min (a), 3 h (b), 6 h (c), and 16 h (d). Normal saline (NS) was employed as carrier. Data were displayed as a boxplot, which indicates the median, interquartile range and the largest and smallest values. Each piece of data represented a group of 6-7 animals. ^*∗*^ *P* < 0.05 compared with the corresponding NS group.

Inhibition of tryptase induced mast cell accumulation by FSLLRY-NH_2_ suggested that the action of tryptase was PAR-2 dependent. In order to confirm activation of PAR-2 is a key event for tryptase induced mast cell accumulation, we investigated effect of agonists of PAR-2 on mast cell accumulation in mice. The results showed that PAR-2 agonist peptide SLIGRL-NH_2_ at the concentration of 50 and 500 *μ*M induced marked mast cell accumulation in peritoneum of mice at 10 min (Figure 3(a)), 3 h (Figure 3(b)), 6 h (Figure 3(c)), and 16 h (Figure 3(d)), and PAR-2 agonist peptide tc-LIGRLO-NH_2_ at the concentration of 50 *μ*M provoked significant mast cell accumulation only at 10 min (Figure 3(a)) and 3 h (Figure 3(b)) following injection. Approximately up to 1.93- and 1.95-fold increases in mast cell numbers were achieved when 500 *μ*M tc-LIGRLO and 500 *μ*M SLIGRL were injected for 10 min and 3 h, respectively (Figures 3(a) and 3(b)). In the same experiments, reverse peptide LRGILS-NH_2_ and tc-OLRGIL-NH_2_ had little effect on accumulation of mast cells.

### 3.5. Effect of Anti-ICAM-1, Anti-L-Selectin, Anti-CD11a, and Anti-CD18 Antibodies on Tryptase Induced Mast Cell Accumulation

**Figure 4:**
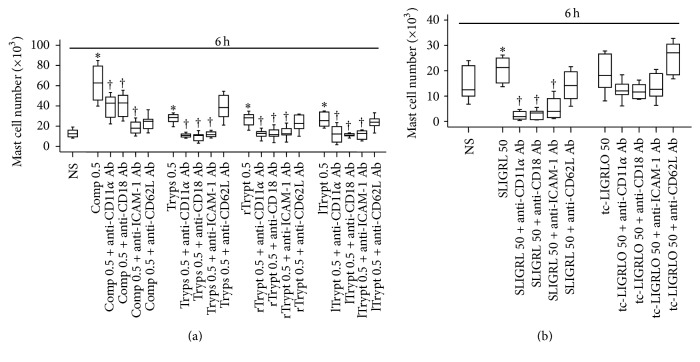
Inhibition of mast cell accumulation by antibodies (Ab) against cell adhesion molecules. Mice were pretreated with monoclonal Ab against L-selectin (anti-CD62L), CD11a (anti-CD11a), CD18 (anti-CD18), and ICAM-1 (anti-ICAM-1), respectively, for 30 min before intraperitoneal injection of (a) compound 48/80 (Comp, *μ*g), lTryptase (lTrypt, *μ*g), or trypsin (Tryps, *μ*g); (b) SLIGRL-NH_2_ (SLIGRL, *μ*M) and tc-LIGRLO-NH_2_ (tc-LIGRLO, *μ*M) for 6 h. Normal saline (NS) was employed as carrier. Data were displayed as a boxplot, which indicates the median, interquartile range and the largest and smallest values. Each piece of data represented a group of 6-7 animals. ^*∗*^ *P* < 0.05 compared with the corresponding NS group. ^†^ *P* < 0.05 compared with the corresponding stimulus alone group.

To understand mechanism of tryptase induced mast cell accumulation, we investigated effect of adhesion molecules on tryptase induced mast cell accumulation in peritoneum of mice. The result showed that anti-CD11a, anti-CD18, and anti-ICAM-1 antibodies suppressed rTryptase, lTryptase, trypsin, and compound 48/80 provoked mast cell accumulation when they were intravenously injected in mice, suggesting that compound 48/80 elicited mast cell accumulation relied at least partially on its capability in induction of mast cell degranulation, and tryptase induced mast cell recruitment was dependent upon CD11a, CD18, and ICAM-1 activities (Figure 4).

### 3.6. Provocation of Serotonin Release in Peritoneum of Mice by Tryptase

**Figure 5:**
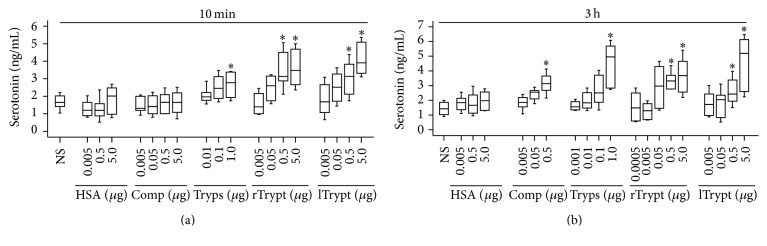
Induction of serotonin release in peritoneum of mice by tryptase. Various concentrations of compound 48/80 (Comp), recombinant tryptase (rTrypt), lung tryptase (lTrypt), and trypsin (Tryps) were injected in peritoneum of mice for 10 min (a) and 3 h (b). Human serum albumin (HSA) was used for foreign protein control, and normal saline (NS) was employed as carrier. Data were displayed as a boxplot, which indicates the median, interquartile range and the largest and smallest values. Each piece of data represented a group of 6-7 animals. ^*∗*^ *P* < 0.05 compared with the corresponding NS group.

Tryptase was reported previously to be capable of inducing mast cell degranulation [6], and serotonin is a marker of mouse mast cell degranulation [18]. We therefore measured concentration of serotonin in peritoneum of mice. The result showed that tryptase induced up to 3.65-fold increase in serotonin concentration in peritoneum of mice as compared with up to 2.2-fold increase induced by compound 48/80 at 3 h following injection (Figure 5(b)). Agonist peptides of PAR-2 appeared to have little effect on concentration of serotonin in mouse peritoneum (data not shown).

### 3.7. Induction of Trans-Endothelium Migration of Mast Cells* In Vitro* by Tryptase

**Figure 6:**
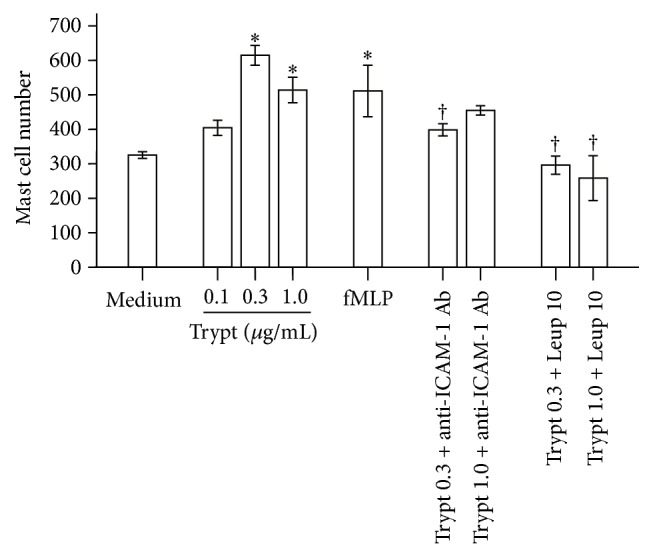
Induction of trans-endothelium migration of mast cells* in vitro* by tryptase. Various concentrations of tryptase (Trypt, *μ*g/mL) with or without leupeptin (Leup, *μ*g/mL) or anti-human ICAM-1 antibody (Ab) were added in chambers of E-16-well plates, respectively, and incubated for 1 h. Cells in the lower chamber were collected for flow cytometric analysis of HMC-1 mast cells. N-formyl-methionyl-leucyl-phenylalanine (fMLP) was used as positive control. Values shown are mean ± SEM for four independent experiments. ^*∗*^ *P* < 0.05 compared with the response to medium alone control. ^†^ *P* < 0.05 compared with the response to the corresponding stimulus alone.

In order to further understand mechanism of mast cell accumulation, we investigated the ability of tryptase in induction of trans-endothelium migration of mast cells* in vitro*. The result showed that tryptase induced up to 89% increase in migration of mast cells. Anti-ICAM-1 antibody and leupeptin completely abolished tryptase induced mast cell migration (Figure 6), suggesting that the action of tryptase was mediated by ICAM-1 and dependent on tryptase enzymatic activity.

## 4. Discussion

The current study demonstrated for the first time that mast cell tryptase is a potent chemoattractant of mast cell accumulation, which may represent a novel self-amplification mechanism of mast cell accumulation. Since mast cell is a pivotal primary effect cell of allergic inflammation and number of mast cells are dramatically increased in the involved allergic tissues [19, 20], our finding not only further emphasizes importance of mast cell major product tryptase in allergy, but also implicates a key mechanism of mast cell accumulation.

We have demonstrated previously that mast cells are able to amplify their own activation-degranulation signals in a paracrine manner [13], which may partially explain the natural fact that a sensitized individual contacts his or her sensitive allergen only once the local allergic inflammation in the involved tissue or organ may last for days or weeks. Since this self-amplification mechanism of mast cell degranulation requires high density of mast cells in involved tissues, investigation of mechanisms of mast cell accumulation becomes priority for mast cell research. The current finding that mast cell tryptase provoked mast cell accumulation in peritoneum of mice explains at least in part how large number of mast cells is accumulated in involved tissue of allergic inflammation.

Numerous mast cell products have been found to be able to induce mast cell migration. Thus, histamine [14] and PAF [15] have been identified as potent chemoattractants of mast cells. Interactions of eotaxin, RANTES, and MCP-1 with CCR3 on mast cells are responsible for the recruitment of these cells [21]. While IL-6 [22] and TNF [23] stimulate migration of mast cells in the presence of laminin, IL-4 induces homotypic aggregation of mast cells in the presence of SCF and IL-6 [24]. Moreover, IL-29 has been found to be released from mast cells and is able to induce mast cell infiltration in mouse peritoneum by a CD18- and ICAM-1-dependent mechanism [25]. Taking together these previously reported findings with our current observation, it is not difficult to understand that mast cell secretory products do have ability to recruit mast cells, which is a self-amplification mechanism of mast cell accumulation.

Three sources of tryptase were used to examine influence of tryptase on mast cell accumulation, and they all possess activity in provocation of mast cell accumulation, suggesting that tryptase induced mast cell accumulation is not a tissue-specific event. It can happen in skin, lung, and likely other tissues. As little as 0.005 and 0.5 *μ*g of skin and lung tryptase were able to induce marked mast cell accumulation, implicating that tryptase is a potent chemoattractant of mast cells. Since large quantities of active form tryptase (up to 35 pg per mast cell) in mast cells [26] and tryptase containing mast cells (MC_T_ type) are predominant subtype of mast cells in lung [27], the above quantity of tryptase is easily achievable in local lung and skin tissues, particularly under allergic conditions. Inhibition of compound 48/80 which is a secretagogue of mast cell degranulation [28] induced mast cell accumulation by inhibitors of tryptase suggests that this synthetic compound induced mast cell accumulation is largely through tryptase.

Inhibition of tryptase induced mast cell accumulation by inhibitors of tryptase indicates that the action of tryptase depends on tryptase enzymatic activity. Since PAR-2 is a substrate of tryptase, antagonist of PAR-2 is able to inhibit tryptase induced mast cell accumulation, and agonists of PAR-2 can also provoke mast cell accumulation in peritoneum of mice, it is very likely that tryptase induced mast cell accumulation is via activation of PAR-2. The observation that PAR-2 may be an important regulator of skin mast cell function during cutaneous inflammation and hypersensitivity [29], that PAR-2 activation through endogenous mast cell tryptase activity could be required to mediate CCL11-induced eosinophil migration [30], and that mast cell tryptase induces eosinophil recruitment in the pleural cavity of mice via PAR-2 [31] may help to understand our current findings.

It was reported that the beta 2-integrin LFA-1 (CD11a/CD18) and the LFA-1/Mac-1 counter-receptor ICAM-1 are expressed on leukaemia (HMC-1 cells) and on normal mature human skin mast cells, which possibly play an important role during homing of immature mast cells as well as during the interaction of activated mast cells with other inflammatory cells [32]. Through CD11a, CD18, and CD54, mature cultured human mast cells can adhere to many extracellular matrix proteins, which may facilitate emigration from the bone marrow into the circulation and ultimately contribute to the tissue homing and localization [33]. Tryptase appears to induce mast cell accumulation in LFA-1 and ICAM-1 dependent pathway in mouse peritoneum. Since ICAM-1 but not the ligands of ICAM-1 is expressed on endothelial cells [34], we anticipate that the interaction between endothelial cell ICAM-1 and mast cell LFA-1 is key for tryptase induced adhesion and trans-endothelial migration of mast cells. Similar finding was observed previously with IL-29 [25].

While trans-endothelial migration mechanism of mast cells remains obscure, our observation demonstrates that ICAM-1 is a key adhesion molecule of tryptase induced mast cell trans-endothelial migration. A previous report that blockage of expression of ICAM-1 inhibited lipopolysaccharide induced processes of firm adhesion and trans-endothelial migration of leucocytes [35] may support our above observation. A PAR-2 mediated mechanism could also be involved in tryptase induced mast cell trans-endothelial migration as inhibitors of tryptase blocked the actions of tryptase on mast cell migration. Involvement of PAR-2 in trans-epithelial migration of neutrophils [36] and upregulation of expression of cell adhesion molecules on neutrophils by agonists of PAR-2 [37] suggest that PAR-2 contributes to migration of neutrophils, which helps to understand our above observation.

Enhanced concentration of serotonin in peritoneum of mice following injection of tryptase suggests that tryptase induced not only mast cell accumulation, but also degranulation as serotonin has been used as a marker of mouse mast cell degranulation [38]. However, serotonin concentration in peritoneum of mice appeared to be not increased in response to agonist peptides of PAR-2, suggesting that tryptase induced mast cell accumulation is via activation of PAR-2, but degranulation is not.

In conclusion, the capability of tryptase in induction of mast cell accumulation implies further that tryptase should be a major proinflammatory mediator in allergy. Provocation of mast cell accumulation by mast cell major granule product suggests a novel self-amplification mechanism of mast cell accumulation in allergic inflammation. Mast cell stabilizer as well as PAR-2 antagonist agents may be useful for treatment of allergic reactions.

## Figures and Tables

**Table 1:** Inhibition of tryptase induced mast cell infiltration by inhibitors of tryptase and heat inactivation of the enzyme at 6 and 16 h following injection.

Treatment	Percentage inhibition of mast cell infiltration	
	6 h	16 h
	Median (range)	Median (range)
Leupeptin 10 *μ*g	91.2 (76.9–100)^*∗*^	81.1 (57.9–100)^*∗*^
Benzamidine 10 *μ*g	84.6 (71.8–100)^*∗*^	71.2 (54.8–100)^*∗*^
Aprotinin 10 *μ*g	82.3 (56.4–100)^*∗*^	87.4 (57.9–100)^*∗*^
Antipain 10 *μ*g	79.5 (61.5–100)^*∗*^	90.1 (62.3–100)^*∗*^
Protamine 10 *μ*g	81.2 (67.7–100)^*∗*^	NA
Heat inactivation 5 *μ*g	90.3 (81.2–100)^*∗*^	91.1 (81.3–100)^*∗*^

The values shown are median (range) for 6–8 individual mice. The inhibitors were incubated with 5 *μ*g of human lung tryptase for 20 min before injection into mouse peritoneum. ^*∗*^ *P* < 0.05 compared with the uninhibited control mice. NA = not available.
